# *Helicobacter pylori* induces cancer cell motility independent of the c-Met receptor

**DOI:** 10.4103/1477-3163.50892

**Published:** 2009-05-06

**Authors:** Jared L. Snider, James A. Cardelli

**Affiliations:** Department of Microbiology and Immunology and the Feist-Weiller Cancer Center, Louisiana State University Health Sciences Center—Shreveport, Shreveport, LA, 71130, USA

**Keywords:** c-Met, CagA, *H. pylori*

## Abstract

**Background:**

The hepatocyte growth factor (HGF) receptor, c-Met, is strongly implicated in late-stage cancer progression and poor patient prognosis. The stomach pathogen, *Helicobacter pylori* (*H. pylori*), was recently proposed to stimulate c-Met phosphorylation dependent upon interaction of c-Met with the bacterial CagA protein required for *H. pylori*-induced cancer cell motility and invasion.

**Materials and Methods:**

In this report, we employed short hairpin RNA (shRNA), western blot analysis using antibodies recognizing phosphorylation at discrete c-Met residues, and immunofluorescence microscopy to investigate the CagA-c-Met interaction.

**Results:**

The data showed that shRNA-mediated c-Met knockdown did not reduce *H. pylori*-induced cell motility, suggesting that c-Met was not required for motility. Surprisingly, c-Met knockdown did not reduce the level of an *H. pylori*-induced protein recognized by a phospho-c-Met antibody. This 125 kD protein was 10 kD smaller than c-Met, suggesting that *H. pylori* did not phosphorylate c-Met but cross-reacted with another protein. This hypothesis was confirmed when c-Met phosphorylation inhibitors did not lower the levels of the bacteria-induced 125 kD protein, and c-Met immunoprecipitation (IP) did not detect this 125 kD protein from *H. pylori*-treated lysates. This protein was identified as a product of antibody cross reactivity with phosphorylated CagA. We also confirmed that CagA interacts with c-Met, but this interaction may have caused previous authors to misinterpret phosphorylated CagA as c-Met phosphorylation. Finally, pretreatment with the proteasomal inhibitor, lactacystin, caused prolonged HGF-induced c-Met phosphorylation and facilitated a CagA-negative *H. pylori* to stimulate AGS cell motility, suggesting that sustained c-Met phosphorylation compensates for the loss of CagA-dependent signaling.

**Conclusions:**

These data demonstrate that *H. pylori* stimulates cancer cell motility independent of the c-Met receptor. We further hypothesize that although *H. pylori* does not target c-Met, the bacteria may still utilize c-Met effector signaling to stimulate CagA-independent cancer cell motility, which may provide a further mechanism of *H. pylori*-dependent gastric cancer progression.

## Introduction

Each year approximately 800,000 people are diagnosed with and 700,000 people die from gastric cancer, the second leading cause of cancer-related deaths worldwide.[1,2] Many factors contribute to the development of this disease, including age, sex, race, and diet, but the largest risk factor is infection with the gram negative, spiral-shaped bacterium, *Helicobacter pylori* (*H. pylori*).[3,4] *H. pylori*-mediated disease progression, including gastritis, mucosal ulceration, and gastric cancer, is dependent upon the bacterial components expressed. The *cag* pathogenicity island (*cag* PAI) contains 32 genes that mostly encode components of a putative type IV secretion system (TFSS), which facilitates transfer of bacterial products into host cells. The CagA protein is also expressed from the *cag* PAI, and is the only known protein to be delivered through the TFSS into epithelial cells.[5] On translocation, CagA is phosphorylated at multiple EPIYA tyrosine phosphorylation domains by two host kinases, Src and Abl.[6–9] CagA then influences intracellular signaling by interacting with proteins that regulate key signaling pathways. Many of these proteins, such as ZO-1, SHP-2, and Grb2, regulate cell adhesion, growth, proliferation, and survival.[10–13]

The MET proto-oncogene encodes c-Met, a transmembrane tyrosine kinase receptor that binds to hepatocyte growth factor (HGF), a potent mitogenic and motogenic factor.[14] Dysregulation of c-Met signaling through overexpression, mutation, or autocrine/paracrine activation, increases the severity of most human cancers, including gastric adenocarcinomas.[15–18] In solid tumors, aberrant c-Met phosphorylation can stimulate epithelial-mesenchymal transition (EMT), a key step in tumor progression to metastatic disease.[18,19] During EMT, stationary tumor cells become motile and invasive, which facilitates migration of these cells into the circulation and dissemination to distant sites in the body.[20–23]

Previous data indicated that cancer cells exhibited a “hummingbird” phenotype indicative of EMT in response to coculture with CagA+ *H. pylori* strains.[24] Churin *et al,* reported that c-Met was phosphorylated in response to coculture with CagA+ *H. pylori*, although the mechanism of c-Met activation was not elucidated.[13] This group also showed that CagA interacts with c-Met in cells cocultured with *H. pylori*, and that this interaction is necessary for the motile phenotype to occur.[13] The data also suggest that *H. pylori*-induced cell invasion requires c-Met activity.[25] These reports reveal that *H. pylori* may not only play a role in gastric carcinogenesis, but also in the progression of the tumor to the later stages of invasion and metastasis through the CagA-dependent activation of the c-Met receptor.

In this study, we investigated the role of c-Met in *H. pylori*-dependent cell motility. Surprisingly, shRNA-mediated c-Met knockdown did not block *H. pylori*-induced cancer cell motility or phosphorylation of a protein 10 kD smaller than c-Met as detected by western blot analysis. Additionally, a c-Met pharmacological inhibitor also failed to block *H. pylori*-stimulated phosphorylation of this 125 kD protein. These data suggest that c-Met is not required for *H. pylori*-induced cell motility, and further experiments led us to conclude that although CagA and c-Met interact, *H. pylori* does not target c-Met activation to induce cancer cell motility. We also provide an explanation of how a previous group misinterpreted phosphorylated CagA for phosphorylated c-Met.

Although c-Met is not targeted by *H. pylori*, we present evidence suggesting that sustained c-Met phosphorylation, which is detected in the majority of gastric adenocarcinomas, can rescue cell motility in response to *H. pylori* CagA-negative strains. Based on this observation, c-Met may still influence *H. pylori*-associated gastric cancer cell motility and tumor progression *in vivo*.

## MATERIALS AND METHODS

### Cell culture and reagents

AGS gastric adenocarcinoma cells (ATCC, Manassas, VA) were cultured in Ham's F-12 as previously described.[26] DU145 prostate cancer cells (ATCC, Manassas, VA) were cultured in RPMI 1640 (Mediatech, Herndon, VA) with identical supplements and under the same conditions as indicated for AGS cells.

The pharmacological inhibitor SU11274 was obtained from EMD Biosciences (San Diego, CA); sodium orthovanadate from Fisher Scientific (Pittsburgh, PA); and lactacystin from Sigma (St. Louis, MO). For experiments, cells were pretreated with inhibitors for one hour (lactacystin, 2.5 *μ*M) or overnight (SU11274, 5 *μ*M) prior to the addition of bacteria. Human recombinant HGF was obtained from EMD Biosciences (San Diego, CA), and used at a concentration of 33 ng/mL.

### Bacterial strains and culture conditions

The *H. pylori* strains, 60190 (ATCC 49503, *cag* PAI^+^, *vacA* s1/m1); 11637 (ATCC 43504, *cag* PAI^+^, *vacA* s1/m1); and Tx30a (ATCC 51932, *cag* PAI^-^, *vacA* s2/m2), were obtained from ATCC (Manassas, VA) and grown on trypticase soy agar plates supplemented with 5% adult defibrinated bovine blood (Gemini, West Sacramento, CA) at 37°C in 5% CO_2_ overnight prior to use in experiments. *H. pylori* mutant strains with disrupted *cagA* (60190Δ*cagA*), *cagE* (60190Δ*cagE*), and *vacA* (60190Δ*vacA*) genes were a kind gift from Dr. Richard Peek (Vanderbilt University, Nashville, TN). These strains were grown on the same plates as wild-type bacteria, but under kanamycin selection (50 *μ*g/mL). Bacteria were passaged daily, and fresh bacteria were thawed on a monthly basis.

### Infection of AGS and DU145 cells and scatter assays

Infection of AGS and DU145 cells and scatter assays were performed as previously described.[26] All microscopy was performed using an Eclipse TE300 inverted microscope (Nikon, Japan) equipped with a mercury lamp for epifluorescent imaging. 10X images were acquired using a CoolSNAP_fx_ monochrome CCD camera (Roper Scientific, Duluth, GA) with IPLab 3.7 for Windows software package (BD, Rockville, MD). Images were then processed with ImageJ software (NIH).

### Western blot analysis

The lysates from cells cocultured with bacteria were collected as previously described.[26] Antibodies specific for tubulin (NeoMarkers, Fremont, CA), CagA (Austral Biologicals, San Ramon, CA), phospho-tyrosine, and phospho-c-Met^Y1234/35^ (Cell Signaling Technology, Beverly, MA), total c-Met (C28, Santa Cruz Biotechnology), or phospho-c-Met^Y1230/34/35^ (Biosource, Camarillo, CA) were used. The membranes were stripped using western *re-probe* (G-Biosciences, St. Louis, MO). Densitometric analysis was performed using ImageJ software (NIH).

### Colloidal gold motility assay

Assay derived from colloidal gold phagokinetic assay as described previously.[26,27]

### Lentiviral delivery of short hairpin RNA (shRNA)

The stable AGS and DU145 cell lines expressing shRNAs and targeting c-Met were generated using MISSION shRNA lentivirus particles (Sigma) according to the manufacturer's protocol, and were briefly described previously.[26] The MISSION shRNA clones selected for optimal expression knockdown in this study were TRCN0000040047 (c-Met; Clone ID: NM_000245.1-4571s1c1; Accession Number NM_000245.1) and SHC002V (nontarget control). The cells expressing the shRNAs targeting GFP were generated previously.[26] Stable cells were cultured in puromycin-containing media (0.6 *μ*g/mL).

### Immunoprecipitations (IPs)

For co-immunoprecipitation (Co-IP) experiments, cells were collected in ice-cold lysis buffer (1% NP-40 in PBS, protease inhibitor cocktail (Roche), 2 mM sodium orthovanadate, 10 *μ*M lactacystin, 10 mM NaF), rocked at 4°C for one hour, and cell debris was pelleted by centrifugation. Conversely, lysates were collected using a more strident lysis buffer (50 mM Tris-HCl, pH 7.5, 5 mM EDTA, 100 mM NaCl, 1% Triton X-100, 0.1% SDS, 10% glycerol, protease inhibitor cocktail (Roche), 2 mM sodium orthovanadate, 10 *μ*M lactacystin, 10 mM NaF) that prevented Co-IP (unpublished data). Supernatants were pre-cleared for one hour with protein A/protein G plus agarose (EMD Biosciences, San Diego, CA) and normal IgG (Santa Cruz Biotechnology, Santa Cruz, CA). Lysates were then incubated with the primary antibody overnight at 4°C. Agarose was added for four hours and pelleted by centrifugation, and pellets and supernatants were boiled in protein loading buffer for 5 min prior to western blot analysis. The antibodies used were CagA (Austral Biologicals, San Ramon, CA) or c-Met (C28, Santa Cruz Biotechnology, Santa Cruz, CA).

## RESULTS

### *H. pylori* induces AGS cell motility independent of c-Met expression

**Figure 1:**
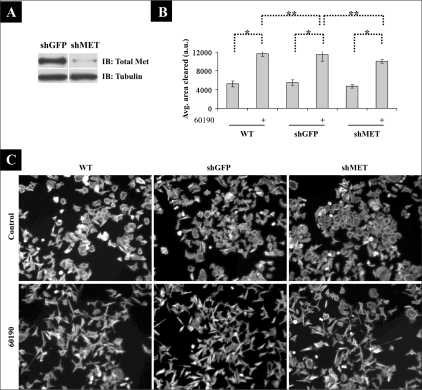
AGS cell c-Met expression knock down does not block H. *pylori*-induced cell morphology changes and motility. (A) AGS cells stably expressing shRNAs against c-Met (shMET) or GFP as a control (shGFP) were generated. Lysates were then analyzed by Western blot analysis for total c-Met protein levels. Tubulin was also probed as a load control. (B) Graph represents the effect of c-Met ablation on H. *pylori*-induced cell motility. Data presented as the area cleared per cell, and compares AGS parental (WT), shGFP, and shMET cells in three separate experiments and includes standard error. *p < 0.001; **p > 0.05. (C) Parental AGS (WT), shGFP, and shMET cells were cultured alone or with 60190 overnight, after which cells were fixed, stained for F-actin, and 10X images were acquired

To investigate the role of c-Met in *H. pylori*-induced cancer cell motility, we generated AGS cells that stably expressed shRNAs targeting c-Met (shMET). These cells exhibited a reduction of 90–95% in c-Met expression compared to AGS cells stably expressing shRNAs targeting GFP (shGFP) as a control [Figure 1A]. Surprisingly, shMET cells responded comparable to shGFP and wild-type (WT) AGS cells by scattering after overnight coculture with *H. pylori* 60190 [Figure 1C]. To determine if c-Met affected *H. pylori*-stimulated cell motility, we applied a colloidal gold motility assay to our overnight coculture experiments.[26] Briefly, cells and bacteria were seeded onto colloidal gold-coated coverslips and incubated overnight, and cell motility was measured as a function of the area that cells cleared during this incubation period. As shown in Figure 1B, we detected no significant change in *H. pylori*-stimulated cell motility in shMET cells compared to WT and shGFP cells. These data suggest that *H. pylori* stimulates cell motility independent of c-Met.

### An *H. pylori*-induced 125 kD phospho-protein is smaller and more sustained in phosphorylation than the HGF-induced 135 kD phospho-c-Met receptor

**Figure 2:**
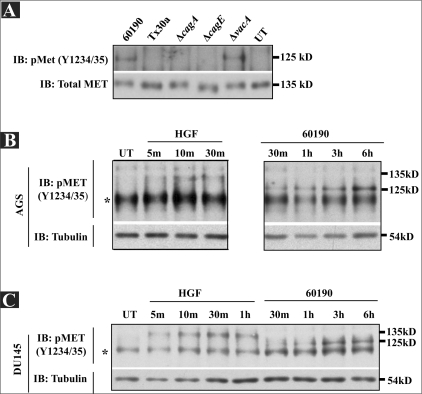
The H. *pylori*-specific band exhibits size and detection kinetics different from HGF-stimulated c-Met phosphorylation. (A) AGS cells were cocultured with the indicated *H. pylori* strains, and lysates were collected after two hours. (B) AGS and (C) DU145 cells were cocultured with 60190 or treated with HGF, and lysates were collected at the indicated time points. Lysates were then analyzed for phospho-c-Met activity by Western blot analysis using a phospho-c-Met (Y1234/35) antibody. Total c-Met (A) or tubulin (B and C) was probed in all blots as a load control. Asterisk indicates nonspecific band

A single report suggested that *H. pylori*-stimulated c-Met phosphorylation in a CagA-dependent manner.[13] To determine if our *H. pylori* strain stimulated c-Met phosphorylation in a CagA-dependent manner, AGS gastric cancer cells were cocultured for two hours with *H. pylori* 60190 (Cag PAI^+^, vacuolating toxin), Tx30a (Cag PAI^-^, nonvacuolating toxin), or isogenic mutants of *H. pylori* 60190 lacking functional *cagA* (60190Δ*cagA*), *cagE* (a key functional gene of the TFSS; 60190Δ*cagE*), or *vacA* (60190Δ*vacA*). Lysates were then collected, and phosphorylation of the c-Met tyrosine kinase domain residues 1234 and 1235, indicative of receptor activation, was detected by Western blot analysis using a commercial antibody specific to phosphorylation at these residues (pMET (Y1234/35); Cell Signaling). As shown in Figure 2A, a protein with a size of approximately 125 kD (10 kD smaller than c-Met running at 135 kDa in Figure 1) was only detected in cells cultured with either the parental 60190 strain or the VacA mutant. The lack of detection of this phosphorylated protein in both 60190Δ*cagA* and 60190Δ*cagE* lysates suggested a requirement for CagA delivery into host cells via the TFSS.

The c-Met receptor migrating at 135 kDa was phosphorylated within 5 min upon addition to HGF in AGS cells; phosphorylation peaked 10 min, and was attenuated over the next 60 min [Figure 2B and Supplemental Figure 1]. While DU145 prostate cancer cells exhibited a longer c-Met activation in response to HGF [Figure 2C], in both DU145 and AGS cells *H. pylori*-induced phosphorylation of the 125 kD protein in a much more sustained fashion [Figures 2B and C]. On the same blot, we also confirmed the size difference between HGF-stimulated phospho-c-Met and the *H. pylori*-induced phospho-protein in both DU145 [Figure 2C] and AGS cells [Figure 4C]. These observations suggest that either 1) *H. pylori* stimulates sustained phosphorylation of a cleaved version of c-Met, or 2) that this 125 kD band detected using a phospho-c-Met-specific antibody is not c-Met. The next section will present data supporting the second possibility.

Supplementary Figure 1 HGF stimulates rapid, but transient, c-Met phosphorylation. AGS cells were treated with HGF, and lysates were collected at the indicated time points. Phospho-c-Met was detected by Western blot analysis using the phospho-c-Met (Y1234/35) antibody. Tubulin was probed as a load control

### Knockdown of c-Met expression significantly lowers HGF-dependent c-Met phosphorylation, but does not lower levels of the *H. pylori*-dependent phospho-125 kD protein

**Figure 3:**
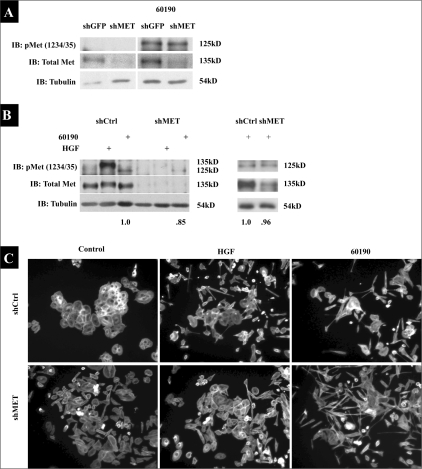
Knock down of c-Met blocks HGF-dependent c-Met activation and cell morphological changes. (A) AGS shMET or shGFP cells were cultured alone or with 60190 for one hour. (B) DU45 cells stably expressing shRNAs targeting c-Met (shMET) or nontarget shRNA (shCtrl) were also generated and were treated with HGF for 15 min or cocultured with 60190 for one hour. All lysates were collected, and phosphorylated and total c-Met were detected by Western blot analysis using phospho-c-Met (Y1234/35) or total c-Met C28 antibodies. Tubulin was probed as a load control, and densitometric analysis was performed, as indicated beneath blots. (C) DU145 shCtrl and shMET cells were cultured alone or with HGF or 60190 overnight, after which cells were fixed, stained for F-actin and 10 X images were acquired

Since AGS shMET cells exhibited no change in *H. pylori*-induced cell morphological changes and motility, these cells were employed to determine if c-Met expression knockdown prevented the appearance of the 125 kD *H. pylori*-induced phospho-protein. The western blot analysis of AGS shMET lysates revealed no change in levels of the phospho-125 kD protein (using the phospho-c-Met (Y1234/35) antibody) as compared to shGFP cells after one hour in the presence of *H. pylori* 60190 [Figure 3A], suggesting that this protein might not be c-Met.

**Figure 4:**
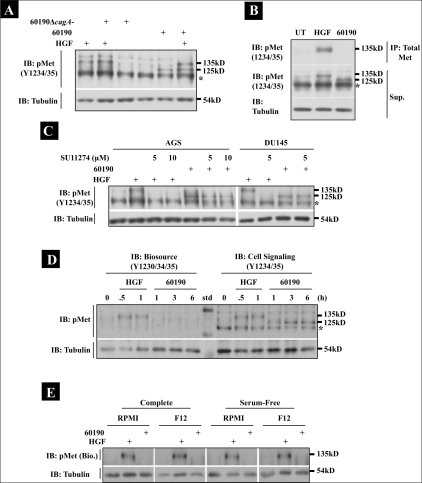
*H. pylori* does not stimulate c-Met phosphorylation. (A) AGS parental cells were cultured alone or with 60190 or 60190Δ*cagA* for one hour. Cells +/− bacteria were then treated with HGF for 15 min. (B) AGS cells were cultured alone or treated with HGF for 15 min or 60190 for three hours, and c-Met was immunoprecipitated from the lysates using a strident lysis buffer that prevented Co-IP (see Methods). (C) AGS or DU145 parental cells were pretreated with DMSO or SU11274 at the indicated concentrations overnight prior to either treatment with HGF for 15 min or coculture with 60190 for three hours. (A, B, C) Phospho-c-Met was detected by Western blot analysis using phospho-c-Met (Y1234/35) antibody. (D) DU145 cells were treated with HGF or cocultured with 60190, and lysates were collected at the indicated time points. Western blot analysis was then performed using either Biosource (Y1230/34/35) or Cell Signaling (Y1234/35) antibodies to detect phospho-c-Met. (E) AGS parental cells were cultured in RPMI or Ham's F12 media +/− serum, and either treated with HGF for 15 min or *H. pylori* for one hour. Phospho-c-Met was detected by Western blot analysis using the Biosource (Y1230/34/35) antibody. For all blots, tubulin was also probed as a load control. Asterisk indicates nonspecific band

To determine if this result was cell-specific, and to address HGF-mediated signaling in shMET cells, we generated DU145 prostate cancer cells that stably express the shRNAs targeting c-Met (shMET). Figure 3B revealed the presence of the *H. pylori*-dependent 125 kD phospho-protein in shMET DU145 prostate tumor cells. Based on the signal seen for the 125 kD band, one could also argue that we see less of the 125 kD protein in shMET cells. But a second, more representative blot [Figure 3B] and densitometric analyses of both blots in Figure 3B demonstrate that knockdown of c-Met expression does not affect levels of the 125 kD band. Furthermore, HGF caused robust c-Met phosphorylation in shCtrl cells, while shMET cells exhibited almost no phosphorylation of the 135 kD protein in the presence of HGF [Figure 3B]. Finally, both HGF and *H. pylori* 60190 stimulated the morphological changes in shCtrl DU145 cells indicative of motility, while shMET cells did not respond morphologically to HGF, but still scattered in response to *H. pylori* coculture [Figure 3C]. These data demonstrate that these observations are not cell-specific, and further suggest that *H. pylori* neither requires nor stimulates c-Met activation to induce cell motility. The following sections will further demonstrate that the 125 kD phospho-protein is not c-Met, but is in fact phosphorylated CagA that is recognized by a cross reactive antibody generated against phospho-Met.

### *H. pylori* does not stimulate c-Met phosphorylation

To further demonstrate that the *H. pylori* specific 125 kD phospho-protein is not c-Met, AGS parental cells were cocultured with *H. pylori* 60190 or *H. pylori* 60190Δ*cagA* for one hour alone or in the presence of HGF. Lysates were then analyzed by Western blot analysis for c-Met phosphorylation at Y1234/35 [Figure 4A]. The *H. pylori*-induced 125 kD phospho-protein required bacterial production of CagA for detection, as the CagA mutant strain was unable to stimulate phosphorylation of this protein. Furthermore, the addition of HGF to cells cocultured with *H. pylori* 60190 resulted in the detection of both the 135 kD HGF-dependent and the 125 kD *H. pylori*-dependent protein. These data further suggest that the 135 kD phospho-Met is still present in cell exposed to HGF and *H. pylori*, implying the 125 kD protein is not a cleavage product of c-Met.

Additionally, if the 125 kD protein was a truncated form of c-Met, then both the 135 and 125 kD proteins would be identified by IP using a specific c-Met antibody. To test this, AGS cells were pretreated with HGF or *H. pylori*, lysates were collected, and c-Met was immunoprecipitated under conditions that prevented protein Co-IP (see Methods). In Figure 4B, Western blot analysis showed that only the 135 kD phosphorylated c-Met protein was detected, and only in the HGF immunoprecipitate. In contrast, the 125 kD phospho-protein remained in the *H. pylori* supernatant; further suggesting that *H. pylori* does not target the c-Met receptor.

Next, cells were pretreated with the c-Met kinase inhibitor, SU11274, prior to treatment with HGF or *H. pylori*. As shown in Figure 4C, HGF-induced c-Met phosphorylation was completely inhibited in the presence of SU11274 in both AGS and DU145 parental cell lines. In contrast, the *H. pylori*-induced 125 kD phospho-protein was still detected using higher concentrations of the c-Met inhibitor.

### The *H. pylori*-induced phosphorylated 125 kD protein is detected as a result of antibody cross-reactivity.

Our data strongly suggest that the 125 kD phospho-protein is not c-Met, although the protein was detected by Western blot analysis using Cell Signaling antibodies presumed to be specific to phosphorylated amino acids found only in c-Met. To test if the 125 kD protein is a product of cross reactivity, we examined the presence of phosphorylated proteins in HGF- or *H. pylori*-treated lysates by Western blot analysis using another commercially-available phospho-c-Met antibody (Y1230/34/35; Biosource). As shown in Figure 4D, while both antibodies detected an HGF-dependent c-Met phosphorylation band (135 kD band), the *H. pylori*-induced 125 kD band was only detected using the Cell Signaling antibody, suggesting that the antibody is cross reacting with another protein.

Churin, *et al.*, described *H. pylori*-induced c-Met phosphorylation using serum-starved AGS cells cultured in RPMI growth medium.[13] Our inability to detect c-Met activation by the bacterium may be due to the difference in culture conditions, since we culture AGS cells in Ham's F12 medium and do not serum-starve the cells prior to experimentation. To determine if experimental conditions affect *H. pylori*-dependent c-Met phosphorylation, AGS cells cultured in either complete or serum-free RPMI or Ham's F12 were cocultured with *H. pylori* 60190 for one hour, and Western blot analysis was performed to detect phosphorylated c-Met with the Biosource antibody. Figure 4E shows that changes in culture conditions do not promote *H. pylori*-stimulated c-Met phosphorylation, since neither the presence of serum nor the change in growth medium caused detection of c-Met phosphorylation using the phospho-c-Met-specific antibody from Biosource.

### CagA size variation could lead to phosphorylated CagA to be misinterpreted as phosphorylated c-Met

**Figure 5:**
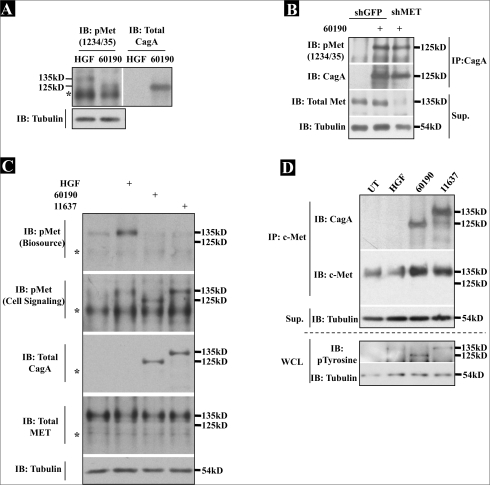
Phosphorylated CagA can be misinterpreted as phospho-c-Met based on variations in CagA size. (A) Lysates of AGS treated with HGF or 60190 were analyzed for phospho-c-Met activity by Western blot analysis using phospho-c-Met (Y1234/35) antibody. Membrane was then stripped and probed for total CagA. (B) AGS shGFP and shMET cells were cultured alone or with 60190 for three hours, and CagA was immunoprecipitated from lysates. Beads and supernatant were then analyzed by Western blot analysis for phosphorylated c-Met (Y1234/35), total c-Met (C28), or CagA using specific antibodies. (C and D) AGS cells were either treated with HGF for 15 min or cocultured with indicated *H. pylori* strains for one hour. (C) Lysates were then analyzed by Western blot analysis for total c-Met, CagA, phospho-tyrosine, or phospho-c-Met using indicated antibodies. (D; upper panel) Total c-Met was immunoprecipitated from indicated lysates, and total CagA and total c-Met, and tubulin were detected in the immunoprecipitates and supernatants by Western blot analysis using specific antibodies. (D; lower panel) Phospho-tyrosine was detected from HGF- or *H. pylori*-treated whole cell lysates (WCL) after 15 min or one hour, respectively. Tubulin was probed in all blots as a load control. Asterisk indicates nonspecific band

Since these data suggest that *H. pylori* does not target c-Met, we next investigated the identity of the 125 kD band detected using the Cell Signaling phospho-c-Met antibodies. Based on our data that the appearance of this phospho-protein was CagA-dependent [Figures 2A and 4A] and similar in size to the CagA protein (125–135 kD), we hypothesized that we were actually detecting phosphorylated CagA. In Figure 5, western blot analysis was performed on lysates from HGF- or *H. pylori*-treated cells using the Cell Signaling phospho-c-Met (Y1234/35)-specific antibody (left panel). Membranes were then stripped and probed for total CagA (right panel). The similar migration observed between the *H. pylori*-dependent 125 kD protein and CagA suggested that the lower molecular weight band detected using the phospho-c-Met antibody (Cell Signaling) is a product of antibody cross-reactivity to CagA. This conclusion was supported by an experiment in which CagA was immunoprecipitated from lysates of AGS shGFP and shMET cells cocultured with *H. pylori* 60190 [Figure 5B]. Western blot analysis showed that equal amounts of the 125 kD phospho-protein was detected using the Cell Signaling antibody in both shGFP and shMET lysates upon addition of *H. pylori*, and this protein correlated in size with the immunoprecipitated CagA.

The CagA protein varies in size, based on the number of EPIYA tyrosine phosphorylation motifs between strains, which in turn influence the ability of CagA to associate with and stimulate host signaling pathways.[28,29] To further investigate if this lower molecular weight band was CagA, AGS cells were treated with HGF for 15 min or cultured with one of the two CagA+ *H. pylori* strains (60190 or 11637) for one hour, and lysates were then analyzed by western blot analysis for c-Met phosphorylation and CagA size. As shown in Figure 5C, c-Met phosphorylation using the Biosource antibody was only detected in response to HGF, while using the Cell Signaling antibody, cells cocultured with *H. pylori* 11637 exhibited a phospho-protein similar in size to the HGF-induced phospho-c-Met band (135 kD) and larger than the 60190-associated band (125 kD). This blot was then stripped and probed for total c-Met, and we detected no change in c-Met size in all lysates, demonstrating that *H. pylori* does not modify the size of c-Met. We also probed for total CagA on these blots, which indicated that the size difference between 60190 and 11637 correlates with the *H. pylori*-associated band size differences detected with the Cell Signaling antibody. These data strongly suggest that the phospho-protein induced by CagA^+^ helicobacter strains is a product of antibody cross-reactivity to the CagA protein, which varies in size between different bacterial strains.

As noted previously, we detect phosphorylated c-Met at specific residues by Western blot analysis of whole cell lysates using antibodies specific to c-Met phosphorylation at discrete residues. The previous authors detected both HGF- and *H. pylori*-dependent c-Met phosphorylation by Western blot analysis of c-Met immunoprecipitates using phospho-tyrosine-specific antibodies.[13,25] Because CagA is also tyrosine phosphorylated, and since we show that CagA can be similar in size to c-Met, we hypothesized that the previous authors actually detected phosphorylated CagA, which was co-immunoprecipitated with c-Met. Indeed, as shown in Figure 5D (upper panel), we show that CagA co-immunoprecipitates with c-Met, confirming that CagA associates with the receptor. However, when we probed whole cell lysates for phosphorylated tyrosine [Figure 5D], we detected a faint 135 kD band indicative of HGF-stimulated phosphorylated c-Met, along with phosphorylated CagA from strain 60190 (125 kD) and 11637 (135 kD). Furthermore, phosphorylated CagA from *H. pylori* 11637 was similar in size to phosphorylated c-Met. Since c-Met and CagA interact, these data suggest that phosphorylated CagA, which co-immunoprecipitates with c-Met, may be wrongly identified as phosphorylated c-Met if the bacterial strain used shows a CagA variant similar in size to c-Met.

### Sustained c-Met activity may influence *H. pylori*-dependent cancer cell motility in a CagA-independent fashion

**Figure 6:**
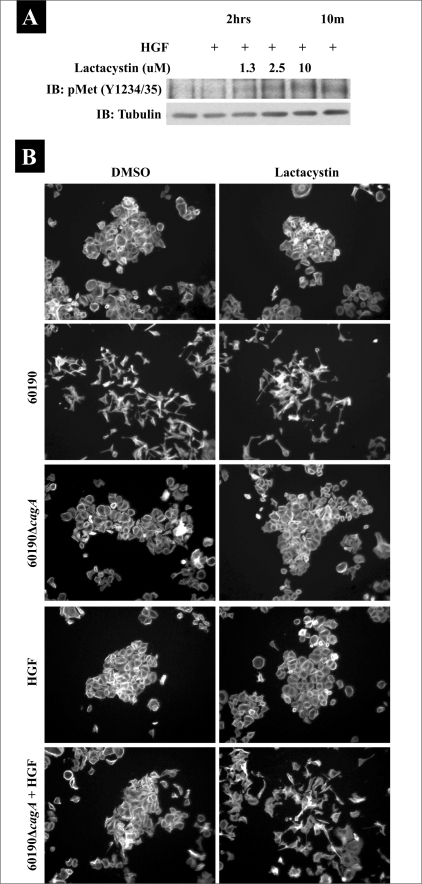
Sustained c-Met phosphorylation rescues CagAindependent cell scattering. AGS parental cells were pretreated with DMSO or lactacystin for two hours, after which lysates were taken 10 min or two hours after the addition of *H. pylori* 60190, *H. pylori* 60190ΔcagA, HGF, or the indicated combinations. Phospho- c-Met was detected by Western blot analysis using the phospho- c-Met (Y1234/35) antibody. Tubulin was probed as a load control. (B) AGS parental cells pretreated with DMSO or lactacystin were cultured alone or treated with HGF, 60190, 60190ΔcagA, or HGF and 60190ΔcagA overnight. Cells were fixed, stained for F-actin, and 10X images were acquired

While we conclude that *H. pylori* neither targets c-Met nor requires receptor activation for cell motility, we propose that *H. pylori* may still utilize c-Met signaling to stimulate cancer cell motility. In DU145 cells, HGF treatment causes transient c-Met phosphorylation and cell scattering indicative of cell motility [Figures 2C and 3C]. AGS cells treated with HGF exhibit an even more transient c-Met activation, which lasts between 5–15 min, and does not stimulate cell scattering [Figures 2B and 6B]. To determine if the extremely transient activation of c-Met is the reason why HGF does not stimulate AGS cell scattering, we first tested if HGF-mediated c-Met phosphorylation could be prolonged in AGS cells or not. Due to the morphogenic/motogenic affects of c-Met activation, receptor phosphorylation is tightly regulated through a combination of phosphatases that directly inactivate the kinase domains and degradation of phosphorylated receptor via the proteosomal degradation pathway.[30–32] Therefore, AGS cells were pretreated with the proteosomal inhibitor, lactacystin, prior to the addition of HGF, and then Western blot analysis was performed to determine if inhibition of c-Met degradation prolonged HGF-induced c-Met phosphorylation in our experimental system [Figure 6A]. Lactacystin alone was insufficient to stimulate c-Met phosphorylation (results not shown), while lactacystin in a dose dependent fashion maintained HGF-induced c-Met phosphorylation at a level seen at 10 min to beyond two hours after addition of HGF.

Since, we could now prolong HGF-induced c-Met phosphorylation in AGS cells, we pretreated cells with DMSO or lactacystin, prior to the addition of HGF, to determine if we could rescue HGF-mediated cell scattering. As shown in Figure 6B, sustained c-Met phosphorylation alone was insufficient to stimulate AGS cell scattering, since cells did not scatter in response to lactacystin alone or in the presence of HGF.

We previously reported that *H. pylori*-induced cell motility occurs through the combination of CagA-dependent and CagA-independent signaling.[26] Therefore, we tested if *H. pylori* CagA-independent signaling complemented sustained c-Met signaling to induce cell scattering. In Figure 6B, while 60190Δ*cagA* alone or with lactacystin was unable to rescue cell scattering, sustained HGF-dependent c-Met phosphorylation by lactacystin treatment facilitated 60190Δ*cagA*-dependent cell scattering. These data suggest that sustained c-Met phosphorylation can complement CagA-independent signaling to stimulate gastric cancer cell motility.

## DISCUSSION

In this report, we demonstrate that *H. pylori* does not require c-Met for induction of cancer cell motility and this bacterium does not stimulate c-Met activation. Furthermore, we demonstrate that this bacterium, in a CagA-dependent fashion, this bacterium, stimulates phosphorylation of a 125 kD protein detected by a phospho-c-Met antibody, but this protein appears to be phospho-CagA and the antibody is cross-reactive. These conclusions are based on the following lines of evidence: 1) shRNA-mediated knockdown of c-Met (greater than 90%) in AGS cells did not prevent *H. pylori*-induced scattering or increases in cell motility; 2) shRNA-mediated c-Met knockdown affected HGF-induced c-Met phosphorylation and morphological changes in DU145 prostate tumor cells indicative of motility, but the loss of 95% of c-Met expression did not affect *H. pylori*-induced scattering and motility; 3) using an antibody generated to phospho-c-Met, an *H. pylori*-dependent phospho-protein was detected by Western blot analysis, but this protein was approximately 10 kD smaller than the band induced by HGF using a phospho-c-Met-specific antibody; 4) the kinetics of phosphorylation of the 125 kD protein was significantly different than HGF-mediated c-Met phosphorylation kinetics; 5) the 125 kD protein was not a truncated form of c-Met, since the band was still detected in shMET cells, and the addition of HGF to *H. pylori*-treated cells produced the HGF-phosphorylated 135 kD protein along with the 125 kD *H. pylori*-induced phosphorylated protein; 6) the c-Met kinase inhibitor, SU11274, failed to prevent *H. pylori*-dependent phosphorylation of the 125 kD protein, but completely inhibited HGF-induced c-Met phosphorylation; 7) c-Met IP from lysates of HGF- or *H. pylori*-treated cells identified the 135 kD phospho-c-Met band in HGF lysates, but not the 125 kD band in *H. pylori* lysates, while IP of CagA recovered the 125 kD phospho-protein; 8) the Biosource phospho-c-Met antibody did not recognize the 125 kD phospho-protein, but recognized HGF-induced phospho-c-Met and 9) the 125 kD *H. pylori*-associated protein correlated with the size of the *H. pylori* 60190 CagA variant. CagA size differs between bacterial strains, and strain 11637 encodes a CagA variant similar in size to c-Met. Consistent with this, Western blot analysis using the Cell Signaling antibody identified a phosphorylated protein in an *H. pylori* 11637 lysate similar in size to HGF-stimulated phospho-c-Met, suggesting that this protein is phospho-CagA and not phospho-c-Met.

Churin *et al*, showed that transient c-Met siRNA transfection of HeLa cells inhibited *H. pylori*-dependent cell scattering[13] and three important issues distinguish our data from the previous report. First, we used AGS gastric carcinoma cells instead of HeLa cervical cancer cells; second, we generated cells that stably expressed shRNAs instead of transient transfection of siRNAs; and third, while the previous report addressed only scattering (which by itself did not seem robust), we performed both scattering and motility assays to determine the role of c-Met in *H. pylori*-induced cell scattering and motility.

The previous authors also detected a phosphorylated protein, similar in size to c-Met, by immunoprecipitating total c-Met and probing for phospho-tyrosine. They concluded that this protein was c-Met, but it is feasible that they were actually detecting phosphorylated CagA migrating at the same molecular weight as c-Met. This group also showed that CagA co-immunoprecipitated with c-Met, a result that we confirmed. We also showed that an *H. pylori* 11637 lysate exhibits a phospho-tyrosine band similar in size to phospho-c-Met in HGF-treated lysates. Since the strain used in the previous study, P1, encodes a CagA variant between 130 and 140 kD in size, it is likely that the authors interpreted phosphorylated CagA, which co-immunoprecipitates with c-Met and is tyrosine-phosphorylated, with phosphorylated c-Met.[33] The report by Oliveira *et al*, also showed that *H. pylori* stimulates c-Met phosphorylation, but the phospho-c-Met band was again detected by IP of total c-Met and phospho-tyrosine immunoblot.[25]

As c-Met plays an important role in the progression of many solid tumors, the report that CagA interacted with and activated c-Met suggested that *H. pylori* could promote gastric cancer progression to metastatic disease. Since our data clearly shows that *H. pylori* does not target c-Met, how can CagA promote cancer cell motility? One hypothesis we tested was that *H. pylori* targeted Ron, a receptor tyrosine kinase closely related to c-Met. By Western blot analysis, though, we concluded that *H. pylori* did not stimulate Ron activation (results not shown). CagA is known to associate with downstream effectors of receptor tyrosine kinase signaling, such as Grb2 and SHP-2, and interaction of CagA with either Grb2 or SHP-2 was required for *H. pylori*-induced cancer cell motility.[10,11] Both of these proteins are recruited to c-Met and bind to the scaffolding adaptor, Grb2-associated binder (Gab) protein, and a recent report demonstrated that CagA expression in *Drosophila* mutants lacking Gab rescued photoreceptor development.[34] These data suggest that CagA may not specifically target a single protein that stimulates cell motility, but may act as an adaptor that recruits multiple signaling molecules.

Another possible CagA target that mediates morphological changes and motility is c-Abl, a nonreceptor tyrosine kinase that interacts with CagA and plays a role in CagA phosphorylation.[8,9] Evidence shows that Src regulates c-Abl activity, which is activated in response to integrin-mediated CagA translocation.[8,9,35,36] Additionally, c-Abl has been shown in recent years to influence cancer invasion and anchorage independence, although the exact mechanism is still unknown.[37–39] Therefore, CagA association with c-Abl may promote cell motility.

Cortactin is an actin-binding protein and a substrate of Src, which represents another possible target that mediates CagA-dependent signaling, necessary for motility. CagA has been shown to dephosphorylate Src, which leads to cortactin deregulation and actin cytoskeletal changes.[33] Therefore, CagA may indirectly influence actin dynamics through cortactin.

Although, we conclude that *H. pylori* does not stimulate c-Met receptor activation, data presented in this report suggest that activated c-Met may allow CagA-negative *H. pylori* strains to induce cancer cell motility. The TFSS was shown recently to interact with the β_1_ integrin, causing activation of the integrin signaling pathway and facilitating CagA translocation and phosphorylation.[35] We previously showed that activation of the β_1_ integrin also leads to downstream activation of JNK and paxillin, both of which are required for *H. pylori*-induced cell motility.[26] These data demonstrate that *H. pylori* stimulates gastric cancer cell motility through a combination of CagA-dependent and CagA-independent signaling. Because increased c-Met activity correlates with late stage cancers and poor patient prognosis, our data that sustained c-Met phosphorylation rescues CagA-negative *H. pylori*-induced cell scattering suggest two important insights into the molecular events that may play a role in *H. pylori*-induced gastric cancer progression. First, since CagA is known to interact with downstream adaptors and effectors of c-Met stimulation, it has been suggested that CagA acts as a docking site to short-circuit c-Met signaling independent of ligand.[34,40] Indeed, our data support this hypothesis, as sustained c-Met activity mimics CagA-dependent signaling to promote cancer cell motility. The second point is that although between 60-70% of *H. pylori* strains are Cag PAI+, the high rate of mutation and infection with multiple strains may allow for not only Cag PAI+, but also CagA-negative strains to promote tumor cell motility.[41] Thus, a greater population of *H. pylori* strains in the stomach may play a role in gastric cancer progression to metastatic disease than previously suggested.

## CONCLUSION

The data from previous reports suggest that *H. pylori* stimulates c-Met phosphorylation in a CagA-dependent manner, and that c-Met is required for *H. pylori*-induced cancer cell motility.[13,25] Employing shRNAs to knockdown c-Met expression, though, we clearly demonstrate that *H. pylori* does not require c-Met to stimulate a motile phenotype in at least two different cell-lines. Furthermore, we show that CagA associates with c-Met, but *H. pylori* does not cause phosphorylation of the receptor. We propose that because CagA is tyrosine phosphorylated and can be similar in size to c-Met, the previous reports misinterpreted phosphorylated CagA as phospho-c-Met based on the published protocol. Although *H. pylori* does not specifically target c-Met, we show that sustained activation of the receptor rescues cell scattering induced by a CagA-negative *H. pylori* mutant (60190Δ*cagA*), suggesting that *H. pylori* utilizes c-Met downstream signaling to stimulate cell motility.
