# Supplementary figures and images for: Helicobacter pylori induces cancer cell motility independent of the c-Met receptor
Source: J Carcinog. 2009 May 6;8:7. doi: 10.4103/1477-3163.50892 (PMC2687142; doi:10.4103/1477-3163.50892)

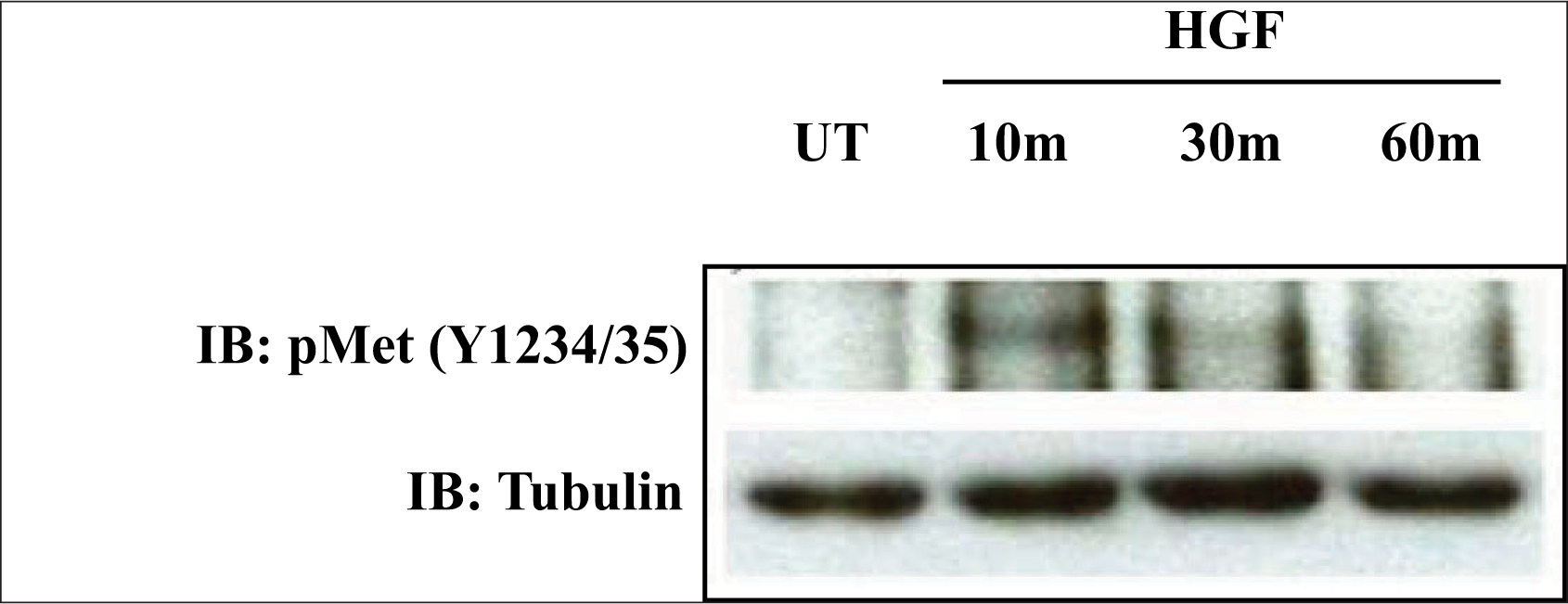

Supplement: Supplementary Figure 1 [file JC-08-50892-g007.tif]
